# Neural Correlates of Hostile Jokes: Cognitive and Motivational Processes in Humor Appreciation

**DOI:** 10.3389/fnhum.2016.00527

**Published:** 2016-10-28

**Authors:** Yu-Chen Chan, Yi-Jun Liao, Cheng-Hao Tu, Hsueh-Chih Chen

**Affiliations:** ^1^Institute of Learning Sciences, National Tsing Hua UniversityHsinchu, Taiwan; ^2^Graduate Institute of Acupuncture Science, China Medical UniversityTaichung, Taiwan; ^3^Department of Educational Psychology and Counseling, National Taiwan Normal UniversityTaipei, Taiwan

**Keywords:** fMRI, aggression, motivational humor, social cognition, mPFC, functional connectivity, superiority theory

## Abstract

Hostile jokes (HJs) provide aggressive catharsis and a feeling of superiority. Behavioral research has found that HJs are perceived as funnier than non-hostile jokes (NJs). The purpose of the present study was to identify the neural correlates of the interaction between type and humor by comparing HJs, NJs, and their corresponding hostile sentences (HSs) and non-hostile sentences (NSs). HJs primarily showed activation in the dorsomedial prefrontal cortex (dmPFC) and midbrain compared with the corresponding hostile baseline. Conversely, NJs primarily revealed activation in the ventromedial PFC (vmPFC), amygdala, midbrain, ventral anterior cingulate cortex, and nucleus accumbens (NAcc) compared with the corresponding non-hostile baseline. These results support the critical role of the medial PFC (mPFC) for the neural correlates of social cognition and socio-emotional processing in response to different types of jokes. Moreover, the processing of HJs showed increased activation in the dmPFC, which suggested cognitive operations of social motivation, whereas the processing of NJs displayed increased activation in the vmPFC, which suggested social-affective engagement. HJs versus NJs primarily showed increased activation in the dmPFC and midbrain, whereas NJs versus HJs primarily displayed greater activation in the amygdala and midbrain. The psychophysiological interaction (PPI) analysis demonstrated functional coupling of the dmPFC–dlPFC and midbrain–dmPFC for HJs and functional coupling of the vmPFC–midbrain and amygdala–midbrain–NAcc for NJs. Surprisingly, HJs were not perceived as funnier than NJs. Future studies could further investigate the neural correlates of potentially important traits of high-hostility tendencies in humor appreciation based on the psychoanalytic and superiority theories of humor.

## Introduction

Humor is a form of social motivation and a way of expressing hostility. Humor provides a means to express and achieve aggression because humor is “not to be taken seriously” (Ziv and Gadish, 1990). Humor often involves aggressive content and is used to demonstrate superiority or to elevate social status (Polimeni and Reiss, 2006); however, the neural substrates underlying social cognition and the reactive aggression of humor that represent affective amusement are poorly understood. The present study attempted to identify the neural correlates of socially motivated (hostile) and non-motivated (non-hostile) jokes in humor appreciation.

Previous functional magnetic resonance imaging (fMRI) studies of verbal humor focused on segregating cognitive and affective processing (e.g., Goel and Dolan, 2001; Chan et al., 2012, 2013). Recent fMRI studies of verbal humor examined the neural correlates of particular humor structures (i.e., logical mechanisms or humor techniques), such as responses to different types of verbal jokes (Chan and Lavallee, 2015), and the processing of different humor structures between the sexes/genders (Chan, 2016a). However, the neural correlates of humor appreciation differ not only in terms of the humor structure (Chan and Lavallee, 2015; Chan, 2016a), but also in terms of humor content (Chan, 2016b).

Behavioral studies of specific types of humor content that contain either sexual or aggressive content have been a topic of interest for years (Lundell, 1993; Crawford, 1995). However, many behavioral studies of humor content have primarily focused on sexual humor (e.g., Hassett and Houlihan, 1979). To date, there has been less research on the neural correlates of humor content differences, specifically pertaining to hostile aggressive content. Therefore, the neural correlates of social motivation on hostility expressed in the underlying humor content of hostile jokes (HJs) should be further investigated.

Hostile humor is hostile aggression in which a target is humiliated, insulted, embarrassed, or physically hurt (Weinstein et al., 2011). HJs are disguised as aggressiveness or veiled attacks that allow one individual to subjugate and conquer another while maintaining respectability and goodness (Freud, 1905/1960). Hostile humor conceptually overlaps with disparagement humor; however, disparagement humor involves humiliation and insult and is directed toward a particular group (Ford and Ferguson, 2004). We define HJs as a sarcastic expression of aggression. HJs (or aggressive jokes) refer to remarks intended to elicit amusement through the denigration of a target. Therefore, HJs employ aggressive content to construct unexpected incongruities that can be resolved in an amusing way through both diminishing and reinterpreting the target.

Evolutionary humor theories have emphasized the possible adaptive characteristics of humor and laughter (Weisfeld, 1993; Polimeni and Reiss, 2006). There have been numerous theoretical links between humor and aggression in humor appreciation. Humor and aggression in humor appreciation have been most influential in social motivation theories, such as the psychoanalytic, superiority/disparagement, and arousal theories (McCauley et al., 1983). The Freudian theory of the joke was the first to propose aggression as a motivation of humor. Freud (1905/1960) viewed humor as having a cathartic effect based on psychoanalytical views. Laughter is considered a pleasurable release of excessive aggressive tension. Additionally, unconscious and tendentious humor should be more appreciated because humor is a socially acceptable sublimation of the aggressive response (Ziv and Gadish, 1990). The superiority theory of humor elicits amusement from the glory of superiority or the triumph one feels from the recognition of the shortcomings or misfortunes of others. Specifically, amusement results from the enhancement of self-esteem derived from a “downward social comparison” with others who are perceived to be inferior or disliked (Hobbes, 1651/1996; Morreall, 1983; Gruner, 1997). People using hostile humor may feel superior to others and use humor to establish their self-esteem (Ferguson and Ford, 2008; Weinstein et al., 2011). Hobbes (1651/1996) suggested that people are amused by the disparagement of others because they enhance their self-esteem through a downward social comparison with the target.

Based on the psychoanalytic theory (Freud, 1905/1960) and superiority theory (Hobbes, 1651/1996; Gruner, 1997), HJs tend to be funnier than more harmless types of non-aggressive jokes. Each framework suggests a distinct mechanism that fosters amusement upon exposure to HJs. Appreciation of hostile humor can be a way to aggress with violating social conventions (Zillmann and Bryant, 1980). Appreciation of hostile humor protects the ego while functioning as a form of interpersonal aggression and a process of tension release or drive reduction (Byrne, 1956). Additionally, the appreciation of hostile humor conveys the explicit expression of disparaging others and establishing superiority (Weinstein et al., 2011).

Empirically supported theories of humor, including the psychoanalytic and superiority theories, argue that aggression underlies the enjoyment of hostile humor. Numerous humor appreciation studies investigating hostile humor have shown that aggressive humor is funnier. Several studies have also indicated that the Freudian cathartic influence of aggressive humor is funnier (e.g., Gollob and Levine, 1967; Berkowitz, 1970; McCauley et al., 1983). For example, the ratings of humor and aggressiveness are consistent with the Freudian, arousal, and superiority theories of humor, which suggest that the tendency of more aggressive humor appears to be funnier (McCauley et al., 1983). The perceived characteristics of protagonists in an aggressive joke have a significant effect on the humor degree of the joke, and a victim who is thought to deserve hostility elicits more humor (Gutman and Priest, 1969). These results are consistent with the Freudian theory of humor. If a person focuses on the aggressive content by asking for an explanation of the joke, the participant is unable to enjoy the humor. However, when the aggressive joke provides sufficient distraction so that the person does not immediately become fully aware of the aggressive implications, the participant can enjoy the aggressive humor (Gollob and Levine, 1967).

Humor operates through a variety of aggressive content types that generate surprise and then amusement and laughter once the unexpected incongruity of hostility is resolved. The prefrontal cortex (PFC) plays a central role in social cognition and involves perspective-taking skills (Frith and Frith, 1999; Bzdok et al., 2013) and the regulation of emotions, such as aggression (Blair, 2004). The specific social brain region in the PFC is the medial PFC (mPFC) (Grossmann, 2013). The mPFC is widely accepted to play a key role in the neural networks relevant for social cognition and socio-emotional processing, which include retrieving social semantic knowledge, monitoring actions and outcomes, mentalizing (or theory of mind, ToM), and processing affective information (Amodio and Frith, 2006; Bzdok et al., 2013).

The mPFC can be broadly divided into two sections based on the neuroanatomical connections: the dorsomedial PFC (dmPFC) and the ventromedial PFC (vmPFC). Imaging studies of social interaction and emotional control have identified at least two distinct areas within the mPFC that are involved in aggression and its control (Lotze et al., 2007). A study of social reactive aggression showed that the dmPFC seemed to represent cognitive operations related to more intense social interaction processes, which included conflict management and response selection in aggression-provoking situations; conversely, the vmPFC might be involved in affective processes associated with compassion toward the victim (Lotze et al., 2007). Additionally, the dmPFC versus the vmPFC has also been proposed to be functionally dissociable in several ways, including cognitive versus emotional, controlled versus automatic, goal-oriented versus outcome-oriented, explicit versus implicit (Bzdok et al., 2013), monitoring and reflective versus stimulus-driven (Olsson and Ochsner, 2008), and considerate of others versus self-involved (Mitchell et al., 2006; Van Overwalle, 2009; Forbes and Grafman, 2010).

Previous studies of verbal humor and meta-analysis results showed that humor processing recruited a large set of cortical and subcortical brain areas that maintained the cognitive and affective components and the laughter response (Chan et al., 2012, 2013; Vrticka et al., 2013; Chan and Lavallee, 2015; Chan, 2016a,b). Humor comprehension recruits activity in the mPFC and requires the attribution of the mental states, whereas humor appreciation recruits activation in classical reward areas, including the midbrain, nucleus accumbens (NAcc), amygdala, insula, ventral anterior cingulate cortex (vACC), and vmPFC, which suggests increased activity in the mesocorticolimbic dopaminergic brain areas (Mobbs et al., 2003; Vrticka et al., 2013). Furthermore, previous fMRI studies of humor appreciation have shown activation in the cortical and subcortical regions in the ventral system, including the amygdala (Watson et al., 2007; Bekinschtein et al., 2011; Chan et al., 2012; Amir et al., 2013; Chan and Lavallee, 2015; Chan, 2016a), midbrain (Watson et al., 2007; Bekinschtein et al., 2011; Chan, 2016a), insula (Watson et al., 2007; Chan, 2016a), vACC (Watson et al., 2007; Chan and Lavallee, 2015), vmPFC (Goel and Dolan, 2001; Chan et al., 2012), and NAcc (Bekinschtein et al., 2011; Chan, 2016a).

The present event-related fMRI study attempted to advance our understanding of the neural correlates of interactions between type (hostile/non-hostile) and humor (joke/non-joke) during humor processing. We also focused on regions of interest (ROIs) in the mPFC (including subregions in the dmPFC and vmPFC), midbrain, amygdala, insula, vACC, and NAcc. The mPFC plays a key role in modulating limbic reactivity for social cognition and social-affective engagement. We predicted that activity in the dmPFC in response to HJs would yield cognitive regulation of emotion behavior when participants made judgments about the hostile aggressive intentions and emotional states of others. Conversely, we predicted that activity in the vmPFC in response to non-hostile jokes (NJs) would be associated with understanding one’s own feelings and would result in physiological changes that accompanied the emotional responses of hedonic reward. Based on the psychoanalytic theory (Freud, 1905/1960) and superiority theory (Hobbes, 1651/1996), we expected that HJs would show greater activation than NJs in the “feelings of amusement” area of the mesocorticolimbic dopamine system.

## Materials and Methods

### Participants

Twenty-five native Mandarin speakers (14 females) with no history of neurological or psychiatric problems participated in this study. Their ages ranged from 20 to 29 years (*M* = 23.56, *SD* = 3.20). Right-hand dominance was indicated by the Edinburgh Handedness Inventory (Oldfield, 1971). The participants’ personalities and senses of humor were evaluated using the traditional Chinese version of the PhoPhiKat-45 (Chen et al., 2011) and the Humor Style Questionnaire (HSQ) (Chan et al., 2011). The mean “katagelasticism” (enjoyment of laughing at others) rating was 1.85 ± 0.38 for the PhoPhiKat-45 on a 4-point scale, and the mean “aggressive humor style” rating was 2.66 ± 0.82 for the HSQ on a 7-point scale. Thus, the participants exhibited low-hostility personality traits. Most participants in this study took part in the experiment reported in Chan (2016b). This study was approved by the Research Ethics Committee of National Tsing Hua University. All participants provided written informed consent in accordance with the Declaration of Helsinki.

### Stimuli

Verbal jokes were assessed as a function of the type of humor. Two types of verbal jokes were presented: hostile and non-hostile. The HJs were selected with aggressive themes, including references to mock-aggressive appearance (e.g., face or shape) and ability, which make fun of a target or victim. The criteria for selecting the stimuli were described in more detail in Chan (2016b).

In the first behavioral study, 44 HJs and 32 NJs were selected from an existing joke corpus taken from previous studies (Cheng et al., 2013; Chan, 2014; Chan and Lavallee, 2015) and the Internet. Fifty-four participants (36 men, mean age 20.41 ± 1.46 years, range 18–24 years) rated each joke on the degree of comprehensibility, funniness, and hostility on a 7-point scale. Thirty-six HJs with ratings above four for comprehensibility, funniness, and hostility were selected. Corresponding baseline trials were constructed by replacing the punch lines of all of the jokes with neutral stories of matching length and punctuation, resulting in 36 hostile sentences (HSs) and 32 non-hostile sentences (NSs).

A second behavioral study was conducted with a separate group of 27 participants (15 men, mean age 20.52 ± 2.41 years, range 18–30 years). Using the E-Prime 2.0 software (Psychological Software Tools, Inc., Pittsburgh, PA, USA), all participants viewed and rated each trial on the degree of comprehensibility, funniness and hostility on a 7-point scale. Based on the results of that study, the present study selected the 32 most salient HJs. The means and standard deviations for the rated levels of comprehensibility, funniness, and hostility for all four conditions of the 32 stimuli were individually investigated. The mean and standard deviation for comprehensibility was 6.29 ± 0.82, which indicated that all stimuli were comprehensible to the participants. The mean funniness ratings for the HJs and NJs were 4.70 ± 1.27 and 5.01 ± 1.17, respectively, compared with mean funniness ratings of 2.82 ± 0.84 and 2.44 ± 0.83 for the HSs and NSs, respectively. The mean hostility ratings for the HJs and HSs were 4.70 ± 1.22 and 4.85 ± 1.09, respectively, compared with the mean non-hostility ratings of 1.61 ± 0.63 and 1.70 ± 0.59 for the NJs and NSs, respectively.

The interaction between type (hostile/non-hostile) and humor (joke/non-joke) in the subjective humor rating was significant at *F*(1,26) = 22.24, *p* < 0.001. The two joke conditions were significantly funnier than the two non-joke conditions. The two hostile conditions (HJ and HS) were rated as being significantly more hostile than the two non-hostile conditions (NJ and NS). The participants recognized the jokes containing hostile intentions.

The hostile jokes (HJs) and baseline sentences (HSs) were constructed using aggressive content. For example, one hostile stimulus pair read as follows:

A highly nearsighted mother brought her child to an art museum to see an art exhibit. Because she was unable to see the portraits clearly, the mother moved her face toward a portrait and shouted, “This is the ugliest portrait I have ever seen in my life.” Suddenly, there was dead silence and nearby tourists said,

“It is not a portrait but a mirror” (HJ) or
“Don’t make so much noise. It’s not classy” (HS).

Non-hostile jokes (NJs) and baseline sentences (NSs) were constructed using non-aggressive content. For example,

A patient was worried about the health of his brain, fearing that he had an incurable disease. He went to the hospital and anxiously asked for a detailed brain scan. After the scan, he asked the doctor, “Is there anything in my brain?” The doctor answered, “Nothing”, and the patient responded,

“That bad?” in a panic (NJ) or
“Thank God!” in relief (NS).

### Experimental Paradigm

The stimuli were presented in an event-related fMRI paradigm. The experimental paradigm was presented using E-Prime, and all stimuli were presented in black and white. The study examined the neural correlates of the interaction between type (hostile and non-hostile) and humor (joke and non-joke). In each trial, the participant was first shown the fixation target for a jittered inter-stimulus interval (ISI), which randomly varied among 2.1, 3.2, 5.6, and 7.9 s and was counterbalanced across the stimulus types. The setup was shown once for 12 s and then followed by the punch line, which lasted for 9 s. Finally, the participants provided a subjective funniness judgment by pressing one of four buttons on a keypad positioned under their right hand to indicate how funny the participant thought the stimuli was (1 = “not funny at all” to 4 = “very funny”), which lasted for 4 s within time-locked rather than self-paced ratings. Instead of paying attention to the judgment process, the present study required participants to appreciate the jokes. Therefore, we designed the instruction as “Please read every short story carefully and appreciate it.” A more detailed account of the experimental design can be viewed in Chan (2016b). There were a total of four functional runs. Each functional run lasted 7 min and 55 s, and there was a 2-min break between runs. The total duration of the experiment was approximately 38 min and 5 s per participant.

### Image Acquisition

Functional MRI data were obtained using a Siemens Skyra 3T scanner (Erlangen, Germany) and a standard 32-channel head coil with a rapid event-related design. Blood oxygenation level-dependent (BOLD) signals were measured with an echo planar imaging (EPI) sequence as follows: repetition time (TR) = 2000 ms, echo time (TE) = 30 ms, flip angle = 90°, 64 × 64 matrix, field of view (FOV) = 240 × 240 mm^2^, and voxel size = 3.75 × 3.75 × 3.7 mm^3^. Each functional run acquired 240 volumes. Every volume contained 36 transverse slices (3.7-mm-thick, no gap) in an interleaved order. A high-resolution 3D structural data set (3D MPRAGE) was acquired using the following pulse sequence: TR = 1900 ms, TE = 3.30 ms, flip angle = 9°, 256 × 256 matrix, FOV = 256 × 256 mm^2^, voxel size = 1 × 1 × 1 mm^3^ resolution, and 192 1-mm thick contiguous axial images.

### Image Analysis

The data were analyzed using the Statistical Parametric Mapping software (SPM8; Wellcome Department of Cognitive Neurology, London, UK). For pre-processing, the EPI data were corrected for the slice time and head movement to the middle functional volume, coregistered, normalized to the standard Montreal Neurological Institute (MNI) coordinate space and spatially smoothed using a Gaussian kernel with a full width at half maximum (FWHM) of 8 mm and high-pass temporal filtering (128 s cutoff).

Statistical maps were generated using a two-level general linear model (GLM) approach. First, each participant’s BOLD signal was modeled with a fixed effects analysis that modeled the different conditions (HJ, HS, NJ, and NS) as events for the punch line using a canonical hemodynamic response function (HRF) with a temporal derivative. All six motion parameters were included as nuisance regressors into the GLM. Each participant was analyzed for his or her responses to the jokes compared with the non-joke baseline stimuli for each condition using a GLM.

Second, each participant’s contrast volumes were fed into a random-effects analysis, which created group average maps for all contrasts across the entire brain using the flexible factorial design. A two-way repeated measures analysis of variance (ANOVA) was performed for predefined jokes and non-jokes, which enabled the evaluation of the main effect of the type (hostile type and non-hostile type), the main effect of humor (joke and non-joke), and the interaction between the two factors (type and humor).

A ROI statistical analysis was performed for a specific a priori hypothesis. Based on social cognition of social motivation (Blair, 2004; Amodio and Frith, 2006; Bzdok et al., 2013; Grossmann, 2013) and previous studies of humor appreciation (Mobbs et al., 2003; Chan et al., 2012, 2013; Vrticka et al., 2013; Chan and Lavallee, 2015; Chan, 2016a,b), the resulting mask of humor processing was associated with brain regions in the predefined ROI using an 8-mm radius sphere. The present study focused on seven ROIs in the PFC (primarily including subregions in the dmPFC and vmPFC), midbrain, amygdala, insula, vACC, and NAcc. Anatomical ROI maps were constructed from the Wake Forest University (WFU) PickAtlas (Maldjian et al., 2003), whereas the PFC (vlPFC, dlPFC, vmPFC, and dmPFC) masks were constructed from the Automated Anatomical Labeling atlas.

A psychophysiological interaction (PPI) analysis (Friston et al., 1997) was also conducted to investigate functional connectivity using the mPFC of social cognition regions, including the left dmPFC in response to HJs and the left vmPFC in response to NJs as the seeds to show connectivity with several regions. In addition to the mPFC for social cognition, the mesocorticolimbic dopamine system, which involves different neural systems that mediate affective amusement behavior, was investigated. Regions of the mesocorticolimbic dopamine system showed an increased response to predictors of a reward. Midbrain and amygdala seed regions were also selected for the PPI analysis. Therefore, individual BOLD signal time courses were extracted from local activation maxima, which served as physiological vectors in the PPI analyses.

The PPI analysis employs three regressors as follows: one regressor representing the deconvolved activation time course in a given volume of interest (the physiological variable; the Y vector), one regressor representing the psychological variable of interest (e.g., joke versus non-joke, including the contrast of HJ versus HS and the contrast of NJ versus NS; the P vector), and a third regressor representing the interaction of the previous two vectors (the PPI term). Using SPM8 for each participant and seed region, we extracted the first eigenvariate of the deconvolved time course of activity in the ROIs identified from an 8-mm-radius sphere around the peak coordinates. The PPI was obtained separately for each participant to assess the neural activity in each of the six predefined ROIs by multiplying the deconvolved and mean-corrected BOLD signal with the psychological vector for the onset times of the joke (1) and non-joke (-1) trials. We computed the PPI by taking the product of the psychological and physiological vectors at each time point.

After convolution with the HRF, mean correction, and orthogonalization, the three regressors (i.e., PPI, Y vector, and P vector) and the effects of no interest (i.e., six motion correction parameters) for each functional run were entered run-by-run into a single first-level GLM to determine condition-dependent changes of functional connectivity for each volume of interest for each participant. The model was estimated and contrasts were generated to test the effect of the PPI used for the second-level random-effects analysis for each ROI. The contrasts were generated to test the effect of the PPI at the second level in one-sample *t*-tests to identify the brain regions showing PPI connectivity with the seed regions.

All reports (including the PPI analyses) of this study were considered significant at *p* < 0.05 after correction for multiple comparisons using the family-wise error rate (FWE) across the ROIs at the voxel level with a cluster size greater than or equal to five voxels after small volume correction on anatomical ROIs. To visualize the signal changes for significant brain regions, time courses were extracted from the beta values of the peak voxels of the regions.

## Results

### Behavioral Data

Participants rated the funniness of each condition on a 4-point scale (1 = not funny at all, 2 = not funny, 3 = funny, and 4 = very funny) during the scanning procedure. The mean funniness ratings for the HJs and NJs were 2.74 ± 0.50 and 2.81 ± 0.51, respectively, compared with mean funniness ratings of 1.97 ± 0.59 and 1.84 ± 0.46 for the HSs and NSs. There was a significant difference in the judged degree of funniness across the four conditions (χ^2^(9) = 372.44, *p* < 0.001). The post hoc tests revealed that the frequency of stimuli rated in the funniness ratings in the joke conditions (HJ and NJ) was significantly higher than the frequency in the non-joke conditions (HS and NS). There was no significant difference in the degree of funniness between the HJ and NJ conditions [*t*(24) = -0.99, *p* = 0.332]. The interaction between the type (hostile and non-hostile) and humor (joke and non-joke) on the funniness ratings was not significant [*F*(1,24) = 3.14, *p* = 0.089, and η^2^ = 0.12]. The main effect of humor (joke/non-joke) was significant [*F*(1,24) = 135.97, *p* < 0.001, and η^2^ = 0.85]. The two joke conditions were significantly funnier than the two non-joke conditions.

### Main Effect and Interaction Analysis

The main effects of type, the main effect of humor and the interaction between type and humor were examined (**Table 1**).

**Table 1:** Main effects of type and humor and their interaction.

Anatomical region	BA	Voxels	Side	Montreal Neurological Institute (MNI) coordinates			*Z*-score
					
				*X*	*y*	*z*	
**Main effect of type: Hostile type (HJ + HS) > Non-hostile type (NJ + NS)**							
Superior frontal gyrus (dmPFC)	8	23	L	-10	54	42	4.37
Medial frontal gyrus (vmPFC)	10	62	L	-6	60	18	4.31
Midbrain	–	18	L	-2	-16	-10	3.59
Nucleus accumbens (NAcc)	–	6	R	6	-6	0	3.29
**Main effect of type: Non-hostile (NJ + NS) > Hostile type (HJ + HS)**							
Middle frontal gyrus (dlPFC)	9	54	R	36	16	36	4.93
Inferior frontal gyrus (vlPFC)	44	37	R	52	16	16	4.57
**Main effect of humor: Joke (HJ + NJ) > non-joke (HS + NS)**							
Medial frontal gyrus (dmPFC)	9	12	L	-8	36	34	4.22
Inferior frontal gyrus (vlPFC)	44	29	R	62	6	20	4.16
Amygdala	–	49	L	-24	-8	-16	4.11
Medial frontal gyrus (vmPFC)	10	61	L	-10	56	4	4.09
Anterior cingulate cortex (vACC)	32	86	L	-8	48	0	4.02
Midbrain	–	56	L	-6	-20	-6	3.93
Superior frontal gyrus (dlPFC)	8	22	L	-18	38	42	3.8
Insula	13	90	L	-38	4	6	3.77
Amygdala	–	12	R	24	-8	-14	3.72
Insula	13	50	R	44	6	4	3.72
**Interaction effect of type × humor**							
Amygdala	–	65	R	26	-8	-14	7.22
Insula	13	81	L	-48	-30	20	6.74
Medial frontal gyrus (dmPFC)	9	47	R	6	50	42	6.12
Anterior cingulate cortex (vACC)	32	76	L	-2	48	-2	6.04
Medial frontal gyrus (vmPFC)	10	39	L	-4	48	-6	5.98
Amygdala	–	48	L	-22	-8	-16	5.82
Inferior frontal gyrus (vlPFC)	44	22	R	62	12	18	4.96
Midbrain	–	14	L	-14	-16	-10	4.76
Midbrain	–	13	R	0	-12	-10	4.67
Inferior frontal gyrus (dlPFC)	9	30	R	46	2	34	4.23


*BA, Brodmann’s area; L, left; R, right; the activation threshold was set at *p* < 0.05 and was FWE (family-wise error rate) corrected for multiple comparisons at the peak level for region-of-interest (ROI) masks. dmPFC, dorsomedial prefrontal cortex; vmPFC, ventromedial PFC; dlPFC, dorsolateral PFC; vlPFC, ventrolateral PFC; vACC, ventral anterior cingulate cortex.*

#### Main Effect of Type (Hostile versus Non-hostile Type)

The contrast of the hostile type (HJ + HS) versus the non-hostile type (NJ + NS) showed greater activation in the left dorsomedial PFC (dmPFC), left ventromedial PFC (vmPFC), left midbrain, and right NAcc (**Table 1**).

#### Main Effect of Type (Non-hostile versus Hostile Type)

The contrast of the non-hostile type (NJ + NS) versus the hostile type (HJ + HS) showed greater activation in the right dorsolateral PFC (dlPFC) and the right ventrolateral PFC (vlPFC) (**Table 1**).

#### Main Effect of Humor (Joke versus Non-joke)

The contrast of all jokes (HJ + NJ) versus all baseline stimuli (HS + NS) showed activation in a wide network of cortical and subcortical regions, including the left dmPFC, right vlPFC, bilateral amygdala, left vmPFC, left ventral anterior cingulate cortex (vACC), left midbrain, left dlPFC, and bilateral insula (**Table 1**).

#### Interaction between Type and Humor

The interaction between type and humor revealed activation in the bilateral amygdala, left insula, right dmPFC, left vACC, left vmPFC, right vlPFC, bilateral midbrain, and right dlPFC (**Table 1**).

### Simple Main Effect for Each Type (Hostile and Non-hostile Type)

A post hoc test showed a significant simple main effect for each of the different types (**Table 2**).

**Table 2:** Simple main effect for ‘type’ in the hostile condition and non-hostile condition and the simple main effect of ‘humor’ in the joke condition.

Anatomical region	BA	Voxels	Side	MNI coordinates			*Z*-score
					
				*x*	*y*	*z*	
**Hostile type (HJ > HS)**							
Middle frontal gyrus (vlPFC)	10	19	L	-44	42	14	4.41
Medial frontal gyrus (dmPFC)	9	7	L	-8	38	34	4.30
Midbrain	–	13	L	-2	-12	-10	3.83
Insula	13	9	L	-34	-8	20	3.59
**Non-hostile type (NJ > NS)**							
Amygdala	–	60	R	24	-8	-14	6.65
Insula	13	214	L	-40	6	4	6.35
Amygdala	–	70	L	-24	-8	-14	6.20
Inferior frontal gyrus (vlPFC)	44	68	R	62	6	18	5.84
Anterior cingulate cortex (vACC)	32	124	L	-4	46	-2	5.83
Medial frontal gyrus (vmPFC)	10	74	L	-6	50	-2	5.82
Superior frontal gyrus (dlPFC)	9	57	L	-20	42	42	5.16
Midbrain	–	146	L	-2	-24	-10	5.07
Midbrain	–	69	L	-14	-16	-4	4.23
Medial frontal gyrus (dmPFC)	9	17	L	-10	40	22	4.23
Insula	13	19	R	46	-18	20	4.11
Medial frontal gyrus (vmPFC)	10	34	R	2	58	-2	4.04
Nucleus accumbens (NAcc)	–	27	R	6	0	0	3.31
**Joke (HJ > NJ)**							
Midbrain	–	35	R	2	-12	-10	4.84
Superior frontal gyrus (dmPFC)	8	38	L	-8	54	40	4.33
Superior frontal gyrus (vlPFC)	10	24	L	-12	62	24	3.87
Insula	13	13	L	-34	-10	22	3.77
**Joke (NJ > HJ)**							
Amygdala	–	59	R	30	-4	-20	5.44
Middle frontal gyrus (vlPFC)	10	21	L	-32	40	22	4.91
Insula	13	98	L	-46	0	0	4.55
Middle frontal gyrus (dlPFC)	9	26	R	36	42	40	4.54
Insula	13	23	R	44	10	14	4.52
Inferior frontal gyrus (vlPFC)	44	18	R	60	16	14	4.24
Middle frontal gyrus (dlPFC)	9	18	L	-36	42	40	4.21
Midbrain	–	14	R	2	-28	-8	3.66
Midbrain	–	13	R	14	-24	-16	3.59


*The activation threshold was set at *p* < 0.05 and was FWE (family wise error rate) corrected.*

#### Hostile Type (HJ > HS)

**FIGURE 1:**
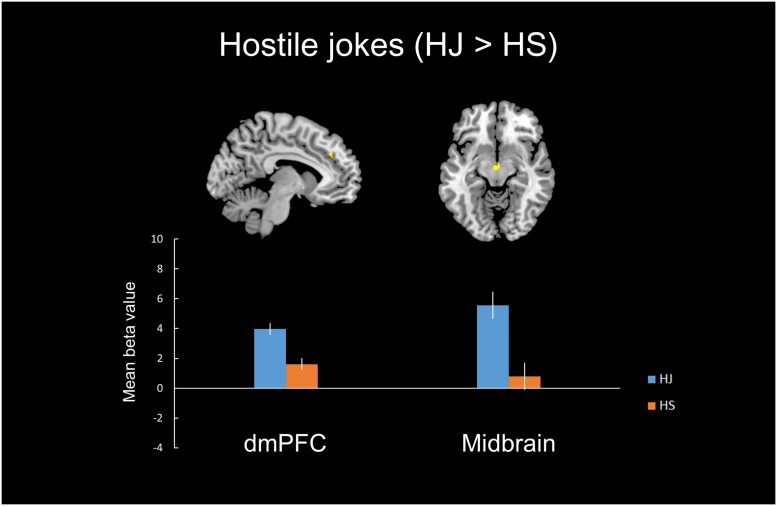
**Distinct neural mechanisms for HJs in the dmPFC.** **(Top)** Brain images of greater activation were found for the simple main effect contrast of hostile jokes with corresponding hostile non-joke baselines (HJ > HS) in the dmPFC during social cognitive processing and in the midbrain during affective amusement processing. **(Bottom)** The bar graphs show the mean beta values of the peak voxels for each of the two types. The error bars represent the SEM. dmPFC, dorsomedial prefrontal cortex; HJ, hostile jokes; HS, hostile baseline stimuli.

In the hostile type condition, the HJ versus HS contrast revealed greater activation in the left vlPFC, left dmPFC, left midbrain, and left insula (**Table 2**, **Figure 1**).

#### Non-hostile Type (NJ > NS)

**FIGURE 2:**
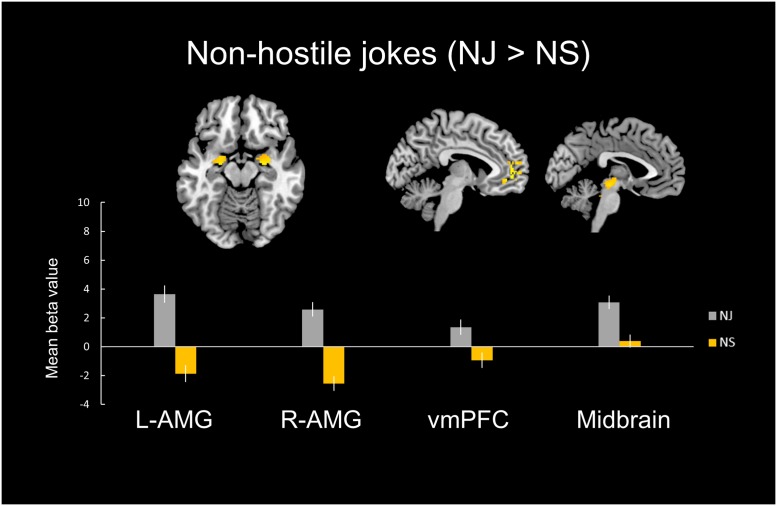
**Distinct neural mechanisms for NJs in the vmPFC.** **(Top)** Brain images of greater activation were found for the simple main effect contrast of non-hostile jokes with the corresponding non-hostile non-joke baseline (HJ > HS) in the vmPFC during socio-emotional processing and in the bilateral amygdala and midbrain during affective amusement processing. **(Bottom)** The bar graphs show the mean beta values of the peak voxels for each of the two types. The error bars represent the SEM. AMG, amygdala; vmPFC, ventromedial prefrontal cortex; NJ, non-hostile jokes; NS, non-hostile baseline stimuli.

In the non-hostile type condition, the NJ versus NS contrast showed greater activation in the bilateral amygdala, bilateral insula, right vlPFC, left vACC, bilateral vmPFC, left dlPFC, left midbrain, left dmPFC, and right NAcc (**Table 2**, **Figure 2**).

### Simple Main Effect of ‘Humor’ in the Joke Condition

#### Joke Condition (HJ > NJ)

In the joke (funny) condition, the HJ versus NJ contrast showed greater activation in the right midbrain, left dmPFC, left vlPFC, and left insula (**Table 2**).

#### Joke Condition (NJ > HJ)

In the joke (funny) condition, the NJ versus HJ contrast showed greater activation in the right amygdala, bilateral vlPFC, bilateral insula, bilateral dlPFC, and right midbrain (**Table 2**).

### Comparison of Two Jokes with Their Corresponding Baselines Removed

#### Hostile versus Non-hostile Type [(HJ-HS) > (NJ-NS)]

A comparison of the two jokes with their corresponding baselines was also performed. In joke condition (HJ versus NJ), represented the humor cognition and appreciation of hostile content. In the hostile versus non-hostile type, indicated the humor appreciation of hostile content. A comparison of neural activation associated with viewing the hostile type (HJ-HS) versus the non-hostile type (NJ-NS) indicated that the hostile type revealed activation in the right dmPFC using a less stringent, uncorrected statistical threshold of *p* < 0.001 (**Table 3**).

**Table 3:** Comparison of the two jokes with their corresponding baselines removed.

Anatomical region	BA	Voxels	Side	MNI coordinates			*Z*-score
					
				*x*	*y*	*z*	
**(HJ–HS) > (NJ–NS)**							
Medial frontal gyrus (dmPFC)^†^	8	14	R	6	36	46	2.91
**(NJ–NS) > (HJ–HS)**							
Amygdala	–	43	R	28	-4	-20	4.82
Insula	13	97	L	-38	6	6	3.93
Amygdala	–	6	L	-24	-8	-16	3.60
Insula	13	24	R	44	-14	-4	3.49
Medial frontal gyrus (vmPFC)	10	31	L	-2	58	0	3.33


*The activation threshold was set at *p* < 0.05 and was FWE corrected.*

*^†^Using a less stringent statistical threshold of *p* < 0.001 that was uncorrected.*

#### Non-hostile versus Hostile Type [(NJ-NS) > (HJ-HS)]

**FIGURE 3:**
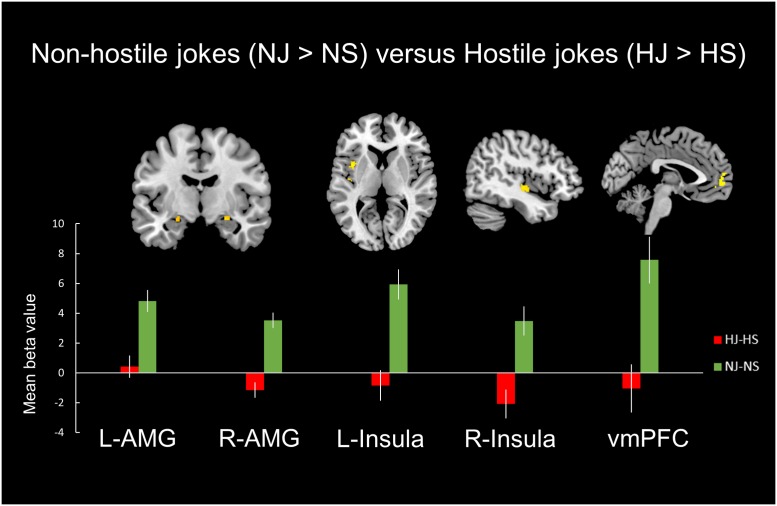
**Distinct neural mechanisms for non-hostile jokes versus hostile jokes in the mesocorticolimbic reward system.** **(Top)** Brain images of greater activation were found for the contrast of non-hostile jokes (NJ-NS) with hostile jokes (HJ-HS) in the bilateral amygdala, bilateral insula, and left vmPFC. **(Bottom)** The bar graphs show the mean beta values of the peak voxels. The error bars represent the SEM. L, left, R, right; AMG, amygdala; vmPFC, ventromedial prefrontal cortex.

A comparison of neural activation associated with viewing the non-hostile type (NJ-NS) versus the hostile type (NJ-NS) revealed significant activation in the bilateral amygdala, bilateral insula, and left vmPFC (**Table 3**, **Figure 3**).

### PPI Analysis

**FIGURE 4:**
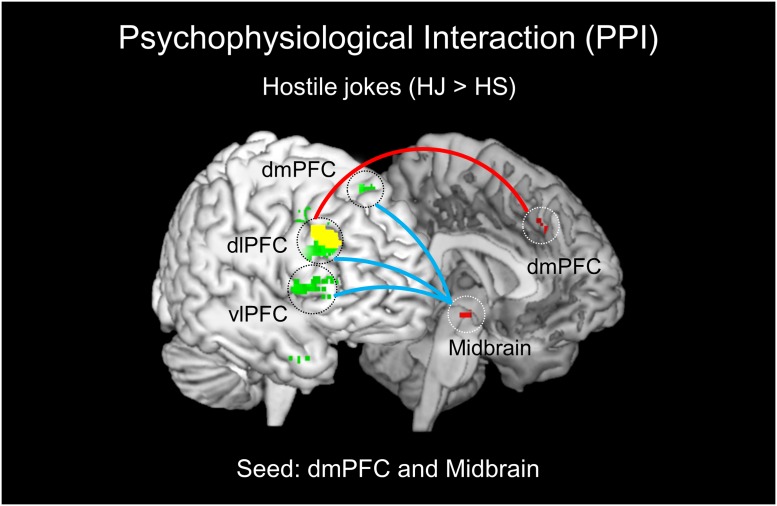
**Results of the psychophysiological interaction (PPI) analysis for HJs.** The left dmPFC and left midbrain were chosen as the seed regions for HJs (red regions). The left dmPFC showed functional connectivity with the right dlPFC (red line). The left midbrain demonstrated functional coupling with the right PFC (dlPFC, dmPFC, and vlPFC) during enjoyment of HJs (blue line).

The PPI analysis revealed a significant interaction between the left dmPFC and hostile joke (HJ > HS) activation that was expressed in regions associated with the social cognition of hostile motivation. The social cognition activity in the dmPFC during hostile jokes (HJ > HS) was accompanied by increased functional interaction with the right dlPFC. Additionally, an increased functional interaction was detected during affective amusement of hostile jokes in the left midbrain with the right dlPFC, dmPFC, and vlPFC (**Table 4**; **Figure 4**).

**Table 4:** Functionalconnectivity of the dmPFC, vmPFC, midbrain, and amygdala seeds of the psychophysiological interaction (PPI) analyses.

Anatomical region	BA	Voxels	Side	MNI coordinates			*Z*-score
					
				*x*	*y*	*z*	
**(I) Hostile jokes**							
**Left dmPFC seed (-8, 38, and 34)**							
Medial frontal gyrus (dlPFC)^†^	9	88	R	42	26	30	3.23
** Left midbrain seed (-2, -12, and -10)**							
Medial frontal gyrus (dlPFC)	46	123	R	44	28	26	4.16
Medial frontal gyrus (dmPFC)	8	104	R	10	24	52	3.77
Medial frontal gyrus (vlPFC)	45	73	R	54	26	4	3.51
**(II) Non-hostile jokes**							
**Left vmPFC seed (-6, 50, and -2)**							
Midbrain	–	56	L	–6	–28	–18	3.26
Midbrain	–	33	L	–8	–24	–14	3.13
** Left midbrain seed (-2, -24, and -10)**							
Medial frontal gyrus (vlPFC)	10	7	L	-24	42	4	3.57
** Left midbrain seed (-14, -16, and -4)**							
Amygdala^†^	–	5	L	-30	-10	-12	2.87
Middle frontal gyrus (dlPFC)^†^	9	5	L	-32	26	40	2.83
** Right amygdala seed (24, -8, and -14)**							
Midbrain	–	48	L	-2	-20	-12	3.73
Nucleus accumbens (NAcc)^†^	–	14	R	10	2	-6	2.89


*The activation threshold was set at *p* < 0.05 and was FWE (family wise error rate) corrected.*

*^†^Using a less stringent statistical threshold of *p* < 0.001 that was uncorrected for ROI comparison.*

**FIGURE 5:**
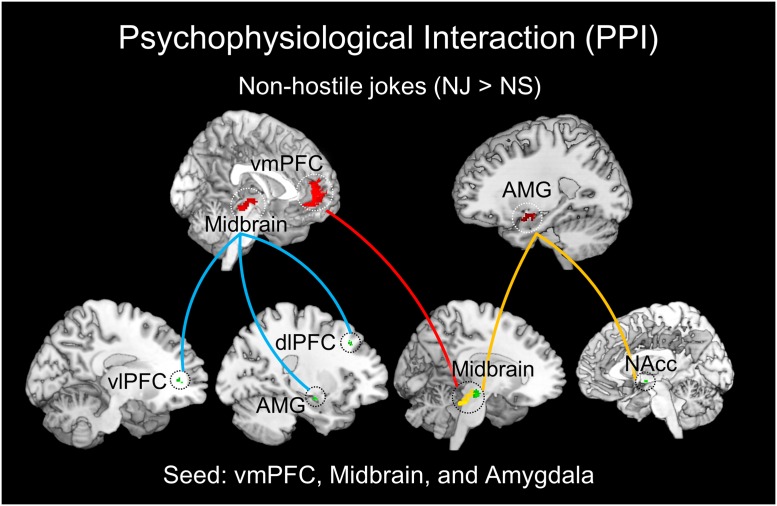
**Results of the PPI analysis for NJs.** The left vmPFC, left midbrain, and right amygdala were chosen as the seed regions for NJs (red regions). The left vmPFC demonstrated functional connectivity with the left midbrain (red line). The left midbrain showed functional connectivity with the left vlPFC, left amygdala, and left dlPFC (blue line). The right amygdala showed functional connectivity with the left midbrain and right nucleus accumbens (NAcc) (yellow line).

The PPI analysis also revealed a significant interaction between the left vmPFC and non-hostile joke (NJ > NS) activation, suggesting social-affective engagement during non-hostile jokes. We found that a stronger functional connectivity of the vmPFC with the left midbrain reflected affective amusement during non-hostile jokes. The PPI analysis using the left midbrain as the seed showed functional connectivity with several regions, including the left vlPFC, left amygdala, and left dlPFC. Additionally, using the left amygdala as the seed revealed functional interaction with the left midbrain and right NAcc (**Table 4**; **Figure 5**).

### Subjective Ratings of Funniness

Individual subjective ratings of funniness were used to identify brain regions with significant activity changes. A four-point Likert scale was utilized in the online funniness judgment during the fMRI scanning. We used two ways to reanalyze the subjective ratings: (1) as a covariate and (2) as a criterion for re-grouping the trials.

We re-estimated the model by serving the rating scores as covariates in a flexible factorial model when performing the second-level analysis. In this flexible factorial model, mean funniness rating scores of each participant for the four conditions respectively were added as a covariate. The statistical analysis of the two-way design with the factors of stimulus type and humor was performed accordingly. The results were generally consistent with our original findings using pre-defined conditions and a corrected threshold of *p* < 0.05 using the FWE across the ROIs. For example, in the NJ-NS contrast, similar activation was found in brain regions including vmPFC, amygdala, midbrain, and insula. After eliminating confounds of rating scores, although dmPFC was also activated in the HJ-HS contrast, non-consciously perceived affective amusement did not lead to activation in the midbrain of insula as seen previously. This could result from participants’ more cognitive involvement in rating hostile jokes subjectively, and thus less non-consciously perceived affective amusement, compared with the original results (i.e. without considering the rating scores as covariates). The process of emotional stimuli contains two neural circuits, a cognitive and an affective path. The former is a conscious process, and the latter is a non-conscious one. Many emotional stimuli are processed unconsciously in subcortical structures (Tamietto and de Gelder, 2010).

Based on participants’ subjective funniness ratings, we also performed the reanalysis from the first level to re-group the trials that participants rated 1-2 for joke condition and 3-4 for non-joke condition. The results using an uncorrected threshold of *p* < 0.001 were generally consistent with our original findings using pre-defined conditions as shown in **Table 2**.

## Discussion

The present study used event-related fMRI to distinguish between the neural mechanisms associated with the processing of hostile and non-hostile jokes and their corresponding non-joke baselines. The present study supports the hypothesis that the mPFC plays a key role in the neural networks relevant for social cognition and socio-emotional processing (Amodio and Frith, 2006; Bzdok et al., 2013). HJs primarily showed greater activation in the dmPFC compared with the corresponding hostile baseline, which suggested cognitive operations of social motivation. Conversely, NJs primarily exhibited increased activation in the vmPFC compared with the corresponding non-hostile baseline, which suggested social-affective engagement. Interestingly, HJs were not perceived as funnier than NJs. NJs versus HJs displayed greater activation in the amygdala, insula, and midbrain. PPI analysis further confirmed functional coupling between dmPFC and dlPFC and increasing coupling between midbrain and dmPFC for HJs. However, PPI analysis demonstrated significantly greater function coupling between vmPFC and midbrain for NJs. Moreover, the results showed functional coupling midbrain and amygdala, and increased coupling between amygdala and midbrain and NAcc for NJs.

The incongruities found in HJs are related to explicit aggressive implications that are left to the reader to discover and “appreciate the joke.” Amusement is experienced when the reader resolves the incongruity and enjoys both a sense of “superiority” and a drive-reducing process based on the implied deprecation of an actor in the jocular episode. Comparison of HJs to corresponding hostile non-joke baselines revealed activation in the vlPFC, dmPFC, midbrain, and insula. The processing of HJs involves the ability to comprehend the mentalizing and hostile aggressive intentions and appreciation of social hostile aggression. The contrast of hostile jokes (HJs) and hostile non-jokes (HSs) increased activation in the left dmPFC and left vlPFC, which may imply the comprehension of the hostile intentions (Chester and DeWall, 2015). In addition, the mesolimbic system including the left midbrain and left insula was significantly activated, which could suggest the appreciation of the social hostile aggression (Chan, 2016a).

The incongruities present in NJs comprise non-aggressive content. Comparison of non-hostile jokes with the corresponding non-hostile non-joke baselines revealed activation in the cortical and subcortical regions. The processing of NJs requires the ability to “get the joke” through schema shifting in the PFC, including the vlPFC, vmPFC, dlPFC, and dmPFC, and also requires the reader to induce a wide range of subcortical regions of amusement in ventral mesocorticolimbic dopaminergic brain areas, including the bilateral amygdala, bilateral insula, bilateral midbrain, right NAcc, and left vACC. The results of the present study are consistent with our previous studies of humor. For all joke types, the left dlPFC appeared to support common cognitive mechanisms, whereas the left vACC was associated with affective amusement (Chan and Lavallee, 2015). The cognitive theory-of-mind (ToM), which is involved in understanding the state of intentions of another person, is found in the dmPFC and vlPFC; conversely, affective empathy, which is the ability to feel the emotions of another person, is exhibited in the vmPFC, anterior insula, and dmPFC (Lieberman, 2007).

The results of the present study also demonstrate the presence of disparate mechanisms underlying the social motivation for particular types of jokes. Specifically, we found that the contrast of hostile jokes (HJ-HS) with non-hostile jokes (NJ-NS) elicited activity in an area of social cognition in the right dmPFC using a less stringent uncorrected statistical threshold. Conversely, the contrast of non-hostile jokes (NJ-NS) with hostile jokes (HJ-HS) elicited activity in socio-emotional processing in the left vmPFC, bilateral amygdala and bilateral insula.

We also more precisely identified content type differences of verbal jokes in the prefrontal modulatory regions, particularly in the mPFC, which suggests social cognition and emotion for mental state attribution in understanding, learning, and emotion regulation (Olsson and Ochsner, 2008). Social cognition of the mentalizing network in the mPFC has reliably been reported to be activated during the processing of socio-emotional stimuli (Singer, 2008; Carrington and Bailey, 2009). The dmPFC was activated during HJs, which suggested a goal of understanding other intentions, whereas the vmPFC was activated during NJs, which suggested affective amusement (Goel and Dolan, 2001; Chan et al., 2012).

Consistent with our expectations, HJs showed greater activation in the dmPFC, whereas NJs showed greater activation in the vmPFC. The dmPFC is implicated in the collaborative intentions between two cartoon characters (Walter et al., 2004) that monitor one’s own social responses and monitor the actions, inferences, and representation of others. The dmPFC is also involved in the assessment of the mental state of oneself and others (Gallagher and Frith, 2003; Amodio and Frith, 2006; Bzdok et al., 2013) and in the cognitive regulation of the emotional state of oneself (Ochsner et al., 2004). The vmPFC was associated with social affective amusement (Goel and Dolan, 2001; Chan et al., 2012), outcome knowledge (i.e., making inferences toward the affective and reward regulation of goal achievement) (Krueger et al., 2009), emotional perspective taking, sympathy (Saxe, 2006), general cognition, reward processing, and representation of social motivation (Schilbach et al., 2010).

Hostile jokes tend to be funnier than non-hostile jokes based on the psychoanalytic theory (Freud, 1905/1960) and superiority theory (Hobbes, 1651/1996; Gruner, 1997). Interestingly, HJs were not funnier than NJs in the affective amusement of social motivation. HJs should be funnier than more harmless types of NJs, and the results appear to be inconsistent with many previous behavioral studies of humor. Previous behavioral studies reported that more hostile humor was typically funnier than more harmless types of humor because the hostile humor content was particularly aggressive and clever (Weinstein et al., 2011). The prediction of the Freudian theory and the superiority theory is that HJs should be funnier than NJs, but the prediction was not supported.

Humor allows us to reduce aggressive drives (Freud, 1905/1960). Freud (1905/1960) suggested that appreciation for humor depended on the state of mind. A state of hostility presumably leads to an appreciation of hostile humor. A number of behavioral studies are broadly consistent with these humor theories, which have suggested that more aggressive humor is funnier (e.g., McCauley et al., 1983). However, the behavioral evidence in favor of such theories is far from conclusive (Strickland, 1959; Byrne, 1961). When a person focuses his attention on the aggressive impulses of humor, his inhibitions become mobilized and he is relatively unable to enjoy the humor (Gollob and Levine, 1967). The degree of arousal of aggressive jokes can affect humor appreciation and produce a cathartic effect (Singer, 1968; Mueller and Donnerstein, 1977). Although hostile humor can facilitate emotional catharsis and release tension, aggression arousal does not significantly affect humor appreciation (Singer, 1968).

Importantly, HJs in humor appreciation have also been explained in terms of disposition differences (Zillmann and Cantor, 1976). Weinstein et al. (2011) found that high-hostility participants especially enjoyed hostile humor. The hostility-arousing participants preferred humorous stimuli that were of a hostile and aggressive nature. It has been frequently reported that aggressive participants prefer aggressive humor (Strickland, 1959; Dworkin and Efran, 1967; Lamb, 1968; Singer, 1968; Landy and Mettee, 1969). The recruitment results of the present study favored low-hostility participants. Therefore, HJs may not be funnier in the mesocorticolimbic reward system than those of NJs, and this hypothesis is worthy of further attention. Future studies should also consider the potentially important traits of high-hostility tendencies, sex/gender differences and intergroup factors in humor appreciation of hostile and non-hostile jokes.

A functional connectivity analysis was performed using PPI to focus on the involvement of social motivation in humor processing in response to hostile and non-hostile jokes. Recent studies have suggested the involvement of the mesolimbic regions in the modulation of affective amusement and frontal cortical reactivity (Banks et al., 2007; Shibata et al., 2014). We demonstrated dmPFC-dlPFC coupling specifically during HJs, whereas the vmPFC-midbrain areas exhibited increased coupling during NJs. Our results support our hypothesis and indicate that the dmPFC and dlPFC are involved in comprehending social motivation for HJs, such as semantic understanding of hostile aggression intentions. The dmPFC-dlPFC connectivity for HJs may play an important role in incongruity detection of aggressive content and both language and semantic integration to achieve the goal of incongruity resolution. Additionally, we found greater activation in the mesocorticolimbic dopaminergic reward system for NJs, including the vmPFC and midbrain. The results showed that vmPFC-midbrain connectivity was involved in affective amusement during the enjoyment of NJs (Chan et al., 2012; Chan, 2016a). These results support the critical role of the medial PFC (mPFC) for the neural correlates of social cognition and socio-emotional processing in different types of jokes. Moreover, the left midbrain demonstrated coupling with the right PFC (dlPFC, dmPFC, and vlPFC) during enjoyment of HJs, whereas the left midbrain showed coupling with the left PFC (vlPFC and dlPFC) and amygdala during enjoyment of NJs. Finally, the right amygdala also showed functional coupling with the left midbrain and right NAcc during enjoyment of NJs.

## Conclusion

Our results support the hypothesis of mPFC involvement in social cognition in response to both types of jokes. HJs require hostile aggressive incongruities. Our results showed that the processing of hostile jokes versus hostile non-jokes increased activation in the dmPFC, which suggested cognitive operations related to monitoring aggression-provoking social motivation, whereas the processing of non-hostile jokes versus non-hostile non-jokes showed increased activation in the vmPFC and suggested affective amusement processes.

Surprisingly, HJs are not funnier than NJs based on Freudian and superiority theories of humor. We found that hostile jokes (HJ-HS) versus non-hostile jokes (NJ-NS) showed increased activation in the right dmPFC, whereas non-hostile jokes (NJ-NS) versus hostile jokes (HJ-HS) displayed greater activation in the bilateral amygdala, bilateral insula, and left vmPFC. Future studies should consider the potentially important disposition differences in humor appreciation (Zillmann and Cantor, 1976).

The PPI analysis provides further evidence for the importance of specific mesocorticolimbic dopaminergic reward networks for both types of jokes. The more non-hostile-dependent jokes showed midbrain–amygdala and amygdala–midbrain/NAcc coupling, the more affective amusement was shown. Future studies should focus on understanding the neural correlates of personality traits and the expressed hostile preferences in humor appreciation based on Freudian and superiority theories of humor.

## Author Contributions

Y-CC designed and conducted the experiment, as well as provided the findings and wrote the paper; Y-JL and C-HT analyzed the data; and H-CC provided the findings.

## Conflict of Interest Statement

The authors declare that the research was conducted in the absence of any commercial or financial relationships that could be construed as a potential conflict of interest.
